# Application of Schistosomiasis Consortium for Operational Research and Evaluation Study Findings to Refine Predictive Modeling of *Schistosoma mansoni* and *Schistosoma haematobium* Control in Sub-Saharan Africa

**DOI:** 10.4269/ajtmh.19-0852

**Published:** 2020-05-12

**Authors:** Charles H. King, Nara Yoon, Xiaoxia Wang, Nathan C. Lo, Ramzi Alsallaq, Martial Ndeffo-Mbah, Emily Li, David Gurarie

**Affiliations:** 1 Center for Global Health and Diseases, Case Western Reserve University, Cleveland, Ohio;; 2 Schistosomiasis Consortium for Operational Research and Evaluation, Center for Tropical and Emerging Global Diseases, University of Georgia, Athens, Georgia;; 3 Department of Mathematics, Applied Mathematics and Statistics, Case Western Reserve University, Cleveland, Ohio;; 4 Department of Medicine, University of California, San Francisco, San Francisco, California;; 5 Yale School of Public Health, Yale University, New Haven, Connecticut;; 6 School of Medicine, Case Western Reserve University, Cleveland, Ohio

## Abstract

An essential mission of the Schistosomiasis Consortium for Operational Research and Evaluation (SCORE) was to help inform global health practices related to the control and elimination of schistosomiasis. To provide more accurate, evidence-based projections of the most likely impact of different control interventions, whether implemented alone or in combination, SCORE supported mathematical modeling teams to provide simulations of community-level *Schistosoma* infection outcomes in the setting of real or hypothetical programs implementing multiyear mass drug administration (MDA) for parasite control. These models were calibrated using SCORE experience with *Schistosoma mansoni* and *Schistosoma haematobium* gaining and sustaining control studies, and with data from comparable programs that used community-based or school-based praziquantel MDA in other parts of sub-Saharan Africa. From 2010 to 2019, models were developed and refined, first to project the likely SCORE control outcomes, and later to more accurately reflect impact of MDA across different transmission settings, including the role of snail ecology and the impact of seasonal rainfall on snail abundance. Starting in 2014, SCORE modeling projections were also compared with the models of colleagues in the Neglected Tropical Diseases Modelling Consortium. To explore further possible improvement to program-based control, later simulations examined the cost-effectiveness of combining MDA with environmental snail control, and the utility of early impact assessment to more quickly identify persistent hot spots of transmission. This article provides a nontechnical summary of the 11 SCORE-related modeling projects and provides links to the original open-access articles describing model development and projections relevant to schistosomiasis control policy.

## OVERVIEW

Optimal development of schistosomiasis control policy requires regular reassessment of available evidence, taken both from program experience and from clinical field trials, on the efficacy and effectiveness of interventions, whether implemented alone or in combination. In many areas, rigorous evidence about the success of available interventions has been limited and resources have often been scarce for the performance of organized control trials.

The Schistosomiasis Consortium for Operational Research and Evaluation (SCORE) was funded in 2008 to conduct research that would contribute to improved decision-making in efforts to control and eliminate schistosomiasis.^1^ Although SCORE included several large-scale epidemiologic studies such as the SCORE gaining and sustaining control trials,^2^ it could not practically compare every combination of interventions. As a result, we turned to well-informed modeling projections to assess the impacts of other interventions that we were unable to test in SCORE field trials. Also, given the uncertainties in the available environmental data, we used mathematical models that capture the biology, dynamics, and ecology of *Schistosoma* transmission to simulate the spectrum of possible outcomes of various proposed public health strategies in a broad range of environments (see Supplemental File S1). This exercise has highlighted existing gaps in diagnostics and available social and environmental data and has helped to describe the potential range in expected program outcomes, giving a more realistic appraisal of the probability of reaching program targets for infection prevalence. Where available, we have calibrated the models against community-level data on local demographics, environment, and infection intensity and prevalence and have used these calibrated models to project the program outcomes for a range of possible preventive interventions for periods ranging from 4 to 30 years. Our aim was to provide program managers with very educated guesses about the likely impact of different interventions, allowing them to assess their available options and to select control approaches likely to have the best chance for success in their location setting.

Most of our modeling work has been based on comparison with existing WHO guidelines for schistosomiasis control, with consideration of hypothetical modifications in implementation (e.g., frequency, coverage, inclusion of adult groups, and combined application of different control measures).^3–6^ The WHO is currently in the process of systematically reviewing experience from ongoing schistosomiasis control programs and has convened a panel to formally revise the recommended control guidelines, based on best-available evidence. Thresholds for implementation and the targets for control are likely to be significantly changed under the new guidelines; however, at present, we do not have any specific details to work with. At the same time, the WHO has convened a technical working group to advise on the best means to perform follow-up surveys to determine program impact and assess where and when implementation will need to be modified to achieve overall program goals. These working group recommendations are also not yet ready for sharing. Nevertheless, we expect that we should be able to quickly revise and recalibrate our models whenever new program targets and new survey approaches are formally recommended for implementation, either at the country or subcountry level.

## INITIAL MODEL DEVELOPMENT

The earliest models supported by SCORE in 2010–2011 were based on a “stratified worm burden” (SWB) model of schistosome transmission that had been newly developed at Case Western Reserve University.^6,7^ The new SWB model was chosen for use because it offered improved predictions of program treatment results as compared with the predictions of previously developed dynamic models that were based on modeling population mean worm burden (MWB).^8–12^ The SWB allowed simulation and endpoint prediction of both prevalence and mean intensity of infection. It also could be adapted to include different infection risks and treatment effects between children and adult age subgroups, and could be adjusted for the varied sensitivity and specificity of different antigen testing versus egg-counting diagnostics.^7,13^

The change to the modeling of prevalence outcomes instead of MWB was important for program managers because the 2002 and 2006 WHO implementation guidelines were based on pre-intervention prevalence of *Schistosoma* among school-age children.^14,15^ In addition, its recommended treatment goals were based on the endpoint prevalence of heavy infections (the subset of infections based with high egg count in stool or urine).^16^ These outputs were not available from standard MWB dynamic models of the time. To inform program planning and policy discussions, our goal was to get beyond the qualitative impact estimates of these older models to provide more accurate quantitative predictions of achievable posttreatment results.

An axiom of mathematical modeling is that “A model should be as simple as possible…but not any simpler…” if it is to have useful application to understanding scientific questions and guiding policy. Many earlier MWB models were based on mathematical analysis for solutions of coupled systems of differential equations, but this approach was not readily adapted to calibration against real-world data or to incorporation of higher levels of system complexity within the model. By contrast, our SWB approach used computer-based numerical simulation based on the programmed dynamic inputs to calculate its predicted outputs. This approach also allowed for needed inclusion of uncertainties in the model inputs and their translation into a probability distribution forecast for the most likely range of possible outcomes, rather than just a single-point estimate.

As new data from SCORE studies became available, we were able to refine the SWB model to improve its predictive accuracy and its utility for policy selection.^3,13,17^ Addition of factors to account for aspects of parasite mating frequency improved predictions of transmission because of low-level infections and the chances of ultimate elimination of transmission. Aspects of the snail side of the transmission cycle also proved influential in correctly predicting the persistence of transmission in the face of declining human prevalence after mass drug administration (MDA).^18^ We explored the influence of seasonality in local transmission, particularly as related to snail abundance and the potential impact of well-timed molluscicide-based snail control.^19^ Incorporating the snail components of transmission also allowed us to explore the potential impact and cost-effectiveness of combined MDA and snail control interventions.^5^ Model enhancements further allowed estimation of the epidemiological impact of combined MDA and possible future anti-schistosome vaccine interventions.^20^

Another important finding of the SCORE gaining and sustaining control projects has been the repeated finding of persistent hot spot communities, where infection prevalence and/or infection intensity are not meaningfully reduced by multiyear MDA implementation.^21–23^ This led us to explore the relative importance of human and environmental factors that can affect MDA outcomes. We then used our calibrated models to define the conditions under which adult treatment and even higher school-age coverage levels (> 85%) become necessary to reach WHO-recommended prevalence targets.^4,24,25^

We also experimented in silico with modifications to current WHO implementation strategies, finding that impact assessments performed in Year 3 of a program, which is earlier than currently recommended,^26^ can more rapidly identify communities that are failing to respond to MDA. Early detection, combined with an earlier shift to more intensive control strategies, was found to potentially accelerate attainment of targeted prevalence goals in a shorter time, with fewer rounds of intervention overall.^4^

The following section provides, in chronological order, brief, nontechnical summaries of the 11 mathematical modeling articles that included simulations based on SCORE study design and/or with SCORE results. For details on model parameterization and the underlying assumptions regarding population demographics and baseline prevalence, the reader is recommended to consult these original articles.

### Wang et al (2012)^6^.

This first SCORE modeling project was designed to predict the likely outcomes of praziquantel MDA on local community *Schistosoma* prevalence during a 4- to 10-year implementation of SCORE-like protocols. The SCORE studies would be comparing the impact of community-based versus school-based delivery of praziquantel treatment on differing schedules in *Schistosoma mansoni*– and *Schistosoma haematobium–*endemic settings in Africa.^2^ Because SCORE project data were not yet available, these first predictive simulations were calibrated and validated using data from published school-based and community-level MDA intervention studies of *S. haematobium* control that had been performed within a network of endemic communities in southeastern Kenya.^27–30^ The study’s simulation approach used the authors’ newly developed SWB modeling approach.^7^ Unlike older models, the SWB model allowed immediate estimation of posttreatment infection prevalence, which was the essential outcome metric used by WHO to define its recommended targets for schistosomiasis control. Separate SWB simulations could be run for different age-groups within a community, including typical variations in their MDA uptake (coverage) and response to treatment by age-group. Results for the different age-groups were then combined to project overall community-level impact when following one of the SCORE randomized trials’ assigned multiyear praziquantel MDA delivery strategies.^31^ Projected results for SCORE study interventions could also be compared with those likely to occur with the current standard-of-care, that is, the 2002/2006 WHO-recommended MDA strategies.^14^

In simulating outcomes for endemic villages, MDA coverage was identified to be a critical factor in reaching the chosen target of < 10% *Schistosoma* prevalence following intervention. Programs achieving only 60% school-age coverage were predicted to take more than 7 years to reach the < 10% prevalence goal, whereas programs reaching > 90% coverage were expected to reach that goal much faster. An important aspect of the analysis was to calibrate each community’s potential for “reworming,” that is, the likelihood of a bounce-back in *Schistosoma* infection prevalence after suspension of MDA. Because of the process of rapid reinfection, the five highest risk villages (having initial school-age prevalence > 35%) were predicted never to reach < 10% prevalence in MDA delivery programs having interval holiday years (in which no praziquantel PDA is given), even if they obtained 70% MDA coverage in treatment years.^6^

These findings reinforced our awareness of the need for intervention programs to have long-term (likely multidecade) sustainability and optimal community uptake to attain schistosomiasis control objectives. They also highlighted the prospective long-term benefits of nondrug interventions (such as habitat modification, provision of alternative safe water sites, and snail control) that could reduce a community’s local transmission potential. Early detection of high- and low-transmission communities was considered essential for optimal selection of intervention strategies. For example, MDA coverage and frequency could be intensified in high-risk locations, and interventions could potentially be lessened (but with continued surveillance) in low-risk communities.

### Gurarie and King (2014) (Ref. 17).

The bounce-back phenomenon, aforementioned, raised interest in the necessity of achieving elimination of *Schistosoma* transmission, where possible.^32^ This was due, in part, to a new awareness of the long-term, subclinical health consequences of the chronic inflammation triggered by *Schistosoma* infections.^33^ In 2012, interest in fully preventing infection (or reinfection) by elimination of transmission was included in the WHO’s 2020 Roadmap on Neglected Tropical Diseases.^34^

A critical challenge for models aiming to predict the timing of elimination in helminth control program is to predict the reproductive success of very low numbers of parasites in low-transmission settings. Because schistosomes are dioecious and have obligate sexual reproduction in the human host, it had been postulated that below a certain population density, there would occur a breakpoint event involving faltering chances of mating, after which the parasite population would go locally extinct.^9,35^ If it occurs in nature, the breakpoint has important implications for a control program because if the program can bring infection below the breakpoint level, intervention could possibly be terminated after just a few rounds of MDA.

The second SCORE modeling article examined the effects of adding schistosome mating and density-dependence factors^36^ on the rates of transmission within endemic locations, as predicted by the SWB model and older MWB models. The analysis identified some inherent inconsistencies in the MWB models, in part because the observed “wormy person” aggregation phenomenon,^37^ in which a few individuals carry a disproportionally high number of worms, is likely to be different between children and adults. We found that the existence of a breakpoint was automatic for MWB models that included mating and aggregation. By contrast, the SWB model revealed a more complex picture. For the SWB model, the existence of a breakpoint was highly sensitive to the probability of adult worm mating failures at low worm numbers, which could be caused by an uneven male/female ratio in a given human host, or affected by rates of polygamy among mated worms. Overall, breakpoints could appear in SWB models only under very stringent conditions, including a strong Allée effect (a low-density mating hurdle). In the end, the “breakpoint region” occupied only a narrow slice of the SWB model’s parameter space. This analysis and subsequent SWB work led us to conclude that, in practice, transmission breakpoint effects are highly unlikely to occur in real-world control programs.

### Gurarie et al (2015) (Ref. 3).

The next project involved collaboration with the newly developed NTD Modelling Consortium (NTDMC),^38^ founded in 2014 by the Bill & Melinda Gates Foundation. One of the nine targeted diseases for the NTDMC initiative was schistosomiasis, for which two modeling groups were recruited. An initial project aimed to test each group’s model predictions using newly acquired SCORE data on *S. haematobium* control obtained from its Mozambique “gaining control” study,^39^ in addition to the previously tested Kenya datasets. To offset the variability of prevalence values based on egg detection by standard parasitological testing, our revised SWB now simulated both worm numbers and their likely associated egg outputs. For this extended model, we also addressed the impact of uncertainties in model inputs, creating a range of credible parameter values with the use of a Bayesian calibration methodology^3^ and, for the first time, we reported our outputs in terms of a forecast range of the most likely possible outcomes.

The 2012 WHO roadmap for NTD control^34^ and the WHO strategic plan for schistosomiasis control^16^ provided 2020 implementation targets for measuring program success. In projecting the impact of a typical *S. haematobium* control program between 2012 and 2020, the SWB model predicted that it would take least eight years to reliably drop any community’s overall infection prevalence below 5%, and that this could occur only with continued high coverage (> 80%) among school-age children. To reduce high-risk community prevalence levels below 2%, the models suggested that at least annual MDA with more than 70% community-wide coverage would be required to achieve this target within a decade. Our calibrated models also suggested that because praziquantel is not fully curative and a fraction of the population goes untreated each year, *Schistosoma* reproduction and transmission remains sufficiently strong for human reinfection to continue locally, even in the face of aggressive MDA. This implies that MDA alone is unlikely to achieve elimination of transmission, and that additional nondrug interventions will be needed to attain that goal.

### Gurarie et al (2016) (Ref. 13).

This fourth, more technical article described in detail the development of our more accurate advanced SWB model. It provided support for the inclusion of additional parameters to adjust for imperfections of standard egg-counting diagnostics and included influential environmental and host factors, aforementioned. It included details of the formulas involved and the methods for model calibration and validation.

### Alsallaq et al (2017) (Ref. 20).

Given our somewhat pessimistic findings regarding the chances for elimination of *Schistosoma* transmission using MDA alone,^3^ we began exploration of the potential impact that non-MDA interventions could have on transmission and local prevalence, whether used alone, or in combination with MDA. However, for decades, development of an anti-schistosome vaccine had remained elusive,^40,41^ evidence from mouse and nonhuman primate studies indicated that vaccine-induced immunity, using purified or cloned parasite antigens, could provide partial protection against new challenge infection^42–44^ and could also reduce the number and fecundity of worms for an established infection.^44^ This combination of both protective and therapeutic effects raised the question of whether such a partially effective vaccine could aid in the ultimate elimination of schistosomiasis.

A vaccine-specific deterministic model, developed by CWRU researchers in collaboration with Yale University partners from the NTDMC, was used to explore the range of efficacy needed for a vaccine to be effective during a 20-year simulation scenario in which vaccination was given as part of childhood immunization, or as part of mass vaccination campaigns.^20^ Duration of vaccine effect was also studied for its influence in determining the likelihood for success. We concluded that a vaccine will need to be moderately to highly effective (60–80% reductions in new infections and current worm burden and in current egg outputs). There will also need to be rapid scale-up and high vaccination coverage in repeated rounds of mass vaccination targeting both children and adults, particularly those who individually cause high levels of environmental egg contamination. Compared with an MDA-only program, vaccination combined with MDA was projected to accelerate and prolong MDA program impact by reducing the rate of acquisition of new worms and by reducing egg release from worms that remain in infected individuals. In that role, vaccination could act as a “praziquantel extender,” reducing the need for annual MDA to obtain suppression of prevalence to WHO target levels.^16,20^ This, in turn, would reduce drug pressure on the local *Schistosoma* population, potentially reducing the risk of emergence of drug resistance. Overall, our model found that vaccination with a partially protective vaccine did have the potential to greatly enhance control program impact in high-prevalence areas, but without certainty of elimination of transmission.

### Gurarie et al (2017) (Ref. 19).

In further exploration of how environmental modification could contribute to the effectiveness of programs for schistosomiasis control, we investigated the role of seasonal versus static snail abundance in modifying the force of transmission in *Schistosoma-*endemic areas. For simplicity, past models have usually assumed a constant snail presence in projecting outcomes of intervention. However, many endemic areas are known to experience large fluctuations in habitat suitability and resultant snail abundance related to monsoonal weather patterns.^45^ This article described a resource-limited growth model that could be calibrated to accurately reflect snail abundance patterns in a *S. haematobium–*endemic region in Kenya. Parameter selection was based on an Approximate Bayesian computation scheme, and resulting models could be used to project the impact of intermittent mollusciciding for suppression of snail numbers. The largest impact of mollusciciding was projected to be immediately after the “long rains” in the first quarter of each year, although the net reductions in snail numbers were modest (20%).^19^ However, by itself, strong seasonality is predicted to reduce the overall rates of human-to-snail-to-human transmission, the impact of intermittent snail control in such settings remains uncertain. This article’s findings played a role in developing SCORE’s Côte d’Ivoire seasonal elimination study,^46^ described in detail by Campbell et al.,^47^ in this supplement.

### Truscott et al (2017) (Ref. 48).

The project described in this article was undertaken by the CWRU-SCORE modeling team in collaboration with schistosomiasis modelers at the London Centre for Neglected Tropical Diseases at the Imperial College, London (ICL), as part of the NTDMC.^38^ The goal of this project was to use treatment data from the SCORE gaining control project for *S. haematobium* (located in northern Mozambique) to compare the ability of two independently developed, calibrated mathematical models to predict control program outcomes in the field. The two models differed somewhat in their description of the parasite life stages outside the human host, and in their representation of the dynamics of the parasite and the aggregation of worm burden within affected human populations. After calibration against a selected Mozambican community’s results, the CWRU model estimated a slightly higher worm burden per person than did the ICL model, and it estimated a lower environmental impact of treatment in terms of snail infection rates.^48^ When the two models were used to project outcomes over a 10-year period, the CWRU model predicted much faster bounce-back of infection prevalence and intensity than did the ICL model, suggesting that elimination of transmission by MDA was very unlikely.

When calibration of the models was attempted using the available field data from communities that had received 2 years of school-based praziquantel MDA, a number of problems were uncovered regarding the Mozambique SCORE study data in terms of estimated school-age population size and reported treatment coverage. Projections of post-MDA prevalence outcomes in years 2 and 3 of the program were over-optimistic in Year 2 (ICL) and over-pessimistic in Year 3 (CWRU) when compared with actual data. The poor quality of the Mozambique data was believed to contribute to the models’ discrepancies.^49^

### Lo et al (2018) (Ref. 5).

This project involved collaboration between the SCORE modeling partners and infectious disease specialists and health economists at Stanford University. Its objective was to use formal cost-effectiveness analysis to examine the potential benefits of combining snail control with school-based or community-wide praziquantel MDA to improve overall reduction of *Schistosoma* infection in high-risk and lower risk communities. Transmission dynamics and impact of intervention were modeled using the previously developed SWB model (including a more realistic snail environment), and costs and health impact estimates (in terms of disability adjusted life-years) were taken from previously published literature.^5,50,51^ In formal cost-effectiveness analysis, inclusion of snail control proved to be highly cost-effective in terms of improving the impact of program implementation, even with systematic noncompliance to MDA in a portion of the community. This was true for low-burden settings but was particularly so in high-burden settings when community-wide MDA was combined with regular biannual focal snail control. The article concluded that combined intervention would be especially effective in dealing with persistent hot spots of transmission,^22^ and in locations where MDA treatment uptake is suboptimal. Although snail control does not directly reduce infection prevalence or treat existing disease, its impact in reducing future infections through modification of transmission was found to be an important leveraging factor in maximizing the impact of MDA.

### Toor et al (2018) (Ref. 25).

The NTDMC was asked by BMGF to provide their projections to WHO regarding the likelihood of NTD control programs reaching WHO schistosomiasis morbidity control targets for the years 2020 and 2025. Based on their NTD Roadmap,^34^ and Strategic Plan for 2012–2020,^16^ the target for community “morbidity control” was to reduce the local prevalence of heavy infections to ≤ 5% among school-age children, and the target for “elimination as a public health problem” (EPHP) was to reduce local heavy infection prevalence in school-age children to ≤ 1%.

For the projections presented in the article, the latest models from ICL and from CWRU were calibrated against a 5-year *S. mansoni* control dataset from SCORE’s gaining control study in Kenya, where communities all had > 24% *S. mansoni* starting prevalence among school-age children. In simulations, the impact of WHO-recommended MDA implementation strategies^14,15,26^ were projected for communities with low, moderate, or high endemic prevalence having a broad range of transmission potential. In Year 6, infection status was reassessed, and the schedule of treatments could be maintained or accelerated to improve response. Prevalences of heavy infections in Year 6 and Year 10 were the primary outcome of interest.

The two models predicted that morbidity control and EPHP could be achieved using school-age children treatment with at least 75% coverage in low-prevalence regions. In moderate-prevalence regions, the application of the standard WHO regimen reached morbidity control within 6 years, but getting to EPHP by Year 10 appeared doubtful. Neither goal appeared attainable in high-prevalence areas, even within 10 years.^25^ Increasing school-age children’s coverage to 85% and inclusion of 40% of adults in MDA slightly increased the probability of achieving EPHP, but this was unlikely to be achieved in more than half of the highest risk communities. Doubling treatment frequency increased the chances of reaching EPHP to about 80%, but with substantial increases in cost and logistic requirements. Despite reductions in the number of heavy infections, the projected overall prevalence of infection often remained high, indicating that MDA implementation did not significantly affect local transmission.

In exploring possible options to improve attainment of WHO target goals, expansion from school-based MDA to community-wide MDA was seen as one means to better approach EPHP. However, its impact is sensitive to the number of cases residing in the adult subpopulation, and data for adults are often not captured by routine program monitoring and evaluation.^52^ Efforts to enhance overall MDA uptake, specifically for those persons who are repeatedly missed during drug distribution, were also seen as likely to boost program impact and to reduce the time to EPHP.^53^ The potential impacts of other, nondrug interventions were discussed, but not formally addressed in this article’s model projections.

To the question, “Are we on our way to achieving the 2020 goals for schistosomiasis morbidity control?” the answer is that much depends on local transmission and program performance factors that can blunt the impact of MDA. Persistent hot spots, where standard MDA has not been successful, will no doubt emerge.^22^ These will need adaptive implementation strategies not yet covered by the WHO guidelines.

### Gurarie et al (2018) (Ref. 18).

In revisiting the SWB model to improve its predictive accuracy, we examined the role of our underlying assumptions about the dynamics of snail populations and the process of snail infection. Our original model had incorporated estimates of snail abundance and their likelihood of having patent infection, that is, of their release of infectious cercariae into local water sites. However, most conventional models make the simplifying assumption that the rate of snail infection was a linear function of human infectivity (egg release by humans into the environment). It was evident that standard models were underestimating the persistence of *Schistosoma* infection in higher prevalence areas, so we developed and calibrated a modified SWB model that included a nonlinear force of infection (FOI) for snails, whereby snail infection is more easily achieved and goes to saturation more quickly on exposure to a given level of human-derived egg output. We then performed comparisons of the predictions of two types of model (linear versus nonlinear FOI). There were marked differences in the long-term projections of MDA outcomes, with the nonlinear model predicting that many communities’ transmission factors would be refractory to MDA effects on egg output, meaning that local elimination was very unlikely to occur. In the nonlinear system, the persistence of a relatively small pool of infected humans could exert a disproportionate effect in maintaining local transmission, and this would increase chances of infection prevalence rebound following cessation of MDA intervention.^6^ We concluded that the nonlinear aspect of snail FOI should be included in mathematical modeling to provide a more realistic prediction of program outcomes in all risk settings.

### Li et al (2019) (Ref. 4).

In anticipation of planned revisions to the WHO guidelines for MDA-based schistosomiasis control, the next modeling project examined the potential benefit of earlier assessments of program impact to detect communities that have poor response to standard implementation of praziquantel MDA so that implementation strategy can be modified in a more timely fashion. In consideration of the persistent hot spots (PHS) phenomenon identified in SCORE’s gaining and sustaining studies,^22^ this modeling project simulated MDA outcomes using the latest SWB models,^18^ calibrating them separately against conditions found in lower-, medium-, and higher risk environments. Each model was run multiple times across a range of possible parameter inputs to generate a 95% confidence distribution of likely MDA outcomes. The model ensembles identified a consistent problem with “reworming” after MDA, that is, return of prevalence and higher infection intensities in the year(s) after drug delivery. These models demonstrated a stagnation of treatment impact by Year 3 of the program, which meant that the current WHO strategies for MDA delivery would fail to reach morbidity control targets in more than 80% of locations, even after 10 years of implementation.

We then suggested a more flexible approach to overcome the limited effectiveness of standard strategies and the observed variability in response among locations. If response of a community by Year 3 of the program was suboptimal, MDA frequency could be increased to biannual (twice-a-year) treatment. If prevalence targets were again not met by Year 5, then addition of local snail control was added. Our model projected that local prevalence of heavy infection would be reduced to < 5% in 100% of villages and to < 1% in 54% of villages by Year 7. Although more complex, and initially more costly, this more intensive, modified approach predicted that WHO morbidity control and EPHP targets could be reached more quickly and with greater certainty than current WHO-recommended intervention. This, in turn, has the potential to reduce the total time and resources needed for countries to achieve their public health goals for definitive schistosomiasis control.

## SUMMARY AND RECOMMENDATIONS FOR FUTURE WORK

In confronting older models with new data^54^ from SCORE’s large-scale field studies, it became evident that although standard MWB models could provide *qualitative* projections of the impact of MDA, they often did not provide accurate *quantitative* estimates of the posttreatment prevalence metrics used for monitoring the success of interventions. Use of the SWB model provided such prevalence estimates directly. Through subsequent model modifications to incorporate the effects of relevant influential biological factors for snails and humans, the accuracy of predictions was progressively enhanced. Then, in confronting emerging program outcomes data with our insights from dynamic models, we saw that better data reporting, both from programs and in research, was essential. Accurate information on treatment coverage and on infection prevalence among adult and preschool age-groups could significantly strengthen model precision, and improve the accuracy of predictions. These subpopulations normally go untreated in programs using school-based MDA delivery. Their local presence, along with the presence of children who regularly miss MDA treatment, leaves persistent reservoirs for re-establishment of transmission that will significantly limit the impact of MDA-only programs.

A consistent finding across these modeling studies was that currently recommended MDA schedules will not meet WHO targets in many high- and moderate-risk locations, nor can they lead to local elimination of transmission. The models highlight the very important risk of prevalence bounce-back once MDA is scaled back or ended.^6^ Given the emergence of the PHS during implementation,^22^ it is also apparent that “one size does not fit all” when it comes to managing schistosomiasis control programs. We see the need for new, more extensive guidelines that will allow early adaptation of implementation based on a much earlier impact assessment than is currently the norm. Such adaptive programs should be able to take into consideration the high degree of transmission heterogeneity within known geographic risk zones. In addition, beyond MDA implementation, there should also be guidance on how to provide environmental modification, such as snail control, to interrupt transmission.

In conclusion, as a result of improvements in mathematical modeling of schistosomiasis control, we are now better able to assess the probable long-term impacts of planned interventions, including accelerated MDA, environmental controls, and even antiparasite vaccines. In future work, we will next apply the diverse and abundant epidemiologic data from the SCORE studies to address additional policy-relevant questions to guide ongoing control programs working to control and eliminate schistosomiasis around the globe.

## Supplemental file

Supplemental materials
