# Supplementary material for: Application of Schistosomiasis Consortium for Operational Research and Evaluation Study Findings to Refine Predictive Modeling of Schistosoma mansoni and Schistosoma haematobium Control in Sub-Saharan Africa
Source: Am J Trop Med Hyg. 2020 May 12;103(1 Suppl):97–104. doi: 10.4269/ajtmh.19-0852 (PMC7351296; doi:10.4269/ajtmh.19-0852)
Supplement: Supplementary file 1 [file tpmd190852.SD1.pdf]

File S1: Technical discussion of model development for SCORE projects

Background

SWB approach. Our model combines a SWB (Stratified Worm Burden) approach for the human population and a Susceptible-Exposed-Infected (SEI) setup for snails (Figure A1).

Here we shall briefly review it (further details can be found in [1-4,5]). A single (homogeneous) SWB system consists of worm burden strata $\{h_m\}$ (stratum h_m made of hosts carrying $m\,\Delta w \le w < (m+1)\Delta w$ adult worms); transitions among strata are due to worm accumulation (rate λ), and resolution (death) γ.

$$\begin{array}{ccccccc} \downarrow S_0 & & \downarrow S_1 & & & & \downarrow S_n \\ h_0(t) & \underset{\gamma}{\overset{\lambda}{\rightleftharpoons}} & h_1(t) & \underset{2\gamma}{\overset{\lambda}{\rightleftharpoons}} \ldots & & \underset{n\gamma}{\overset{\lambda}{\rightleftharpoons}} & h_n(t) \\ \downarrow \mu & & \downarrow \mu & & & & \downarrow \mu \end{array} \quad (1)$$

Stratification is determined by worm-step $\Delta w \ge 1$, which serves as a hypothetical *mating threshold*, so the lowest stratum h_0 is assumed infection transmission-free (no mated couples).

Worm mating is an important factor of transmission dynamics. The original paper[1] took mated count approximately equal to the number of adult pairs in each stratum,

$$\phi_m \approx \frac{m\,\Delta w}{2}$$

Subsequent papers refined it, [2-10] following the approach of May,[11] so that the stratum carrying *m* adult worms has

$$\phi_m \approx \frac{m}{2}\left[1 - 2^{-m}\binom{m}{m/2}\right] \quad (2)$$

mated couples. Variables $\{h_m\}$ are normalized ($\sum_{m\ge0} h_m = 1$).

The basic SWB system (1) can be embedded into any demographic (or other risk-behavioral) structure, indicated by the vertical arrows of (1) : $\{S_m\}$ - 'sources', $\{\mu\}$ - turnover rates, due to mortality, maturation, migration, etc. In many applications ([3-10,12]), we divided the human population into 3 age groups, e.g. pre-school children: 1-5 years old, school-aged children (SAC): 6-14 years old, and adults: 15+ years old.

Each demographic group (C- child, S- SAC, A –adult) has its own SWB variables, $\vec{h}^C = (h_0^C, h_1^C, \ldots)$, $\vec{h}^S = (h_0^S, h_1^S, \ldots)$, $\vec{h}^A = (h_0^A, h_1^A, \ldots)$, which obey a coupled system of differential equations described by a transition matrix with age-specific FOI (λ), population turnover (μ), and worm mortality (γ), $M(\lambda, \mu, \gamma)$ as shown below.

$$\frac{d\vec{h}^C}{dt} = M(\lambda_C, \mu_C, \gamma_C)\cdot\vec{h}^C + \vec{S}_C + \mu_C\delta_0$$
$$\frac{d\vec{h}^S}{dt} = M(\lambda_S, \mu_S, \gamma_S)\cdot\vec{h}^S + \mu_S\vec{h}^C \quad (3)$$
$$\frac{d\vec{h}^A}{dt} = M(\lambda_A, \mu_A, \gamma_A)\cdot\vec{h}^A + \mu_A\vec{h}^S$$

Here SWB variables for a younger age (e.g. $\vec{h}^C$) serve as demographic sources for older group (S), and the youngest (children) has its source determined by the human birth rate S_0, $\vec{S}_C = (S_0, 0, 0, ...)$, and all newborn are infection free. Human FOI are determined by snail infectivity, population density, and age-specific contact patterns as explained below.

Human infectivity. Egg release by infected hosts is another important factor in diagnostics and transmission dynamics. Earlier works [1,2,12] assumed steady egg release by SWB-hosts determined by the mean worm fecundity parameter ρ. Hence, we considered test diagnostics and the force of snail infection (FOI) to be functions of *worm burden* distribution (or its *mean*) for a given host group. However, egg release by individual hosts has been shown to be highly irregular and over-dispersed.[13,14]

We adopted these finding to develop a refined model of human infectivity, based on a negative binomial (NB) distribution of egg output for individual hosts and for host strata.[3-5,8,10] Thus, in our setup, egg release by each host is a random process based on NB-distribution. The host stratum carrying $<m>$ adult worms (mean count) has its age-specific mean egg release $E_m = \rho\phi_m$ - a product of age-specific worm fecundity (ρ) and the mated-couple count (2). The NB-aggregation parameter for the m-th stratum is $k_m = k\,\phi_m$. The combined egg release by the entire SWB community/ population group is considered to be a random draw from the resulting NB-mixture distribution across all host strata, weighted via SWB variables $\{h_m\}$

$$\mathcal{D} = \sum_{m\geq 0} h_m\, NB(\rho\phi_m \mid k\,\phi_m) \quad (4)$$

The NB-mixture distribution plays an important role in model calibration and test data analysis, but in the coupled human-snail system (dynamic simulations) we use *mean host infectivity* (egg-release) by each group, is “worm fecundity” x “mean mated count”.

$$E = \langle\mathcal{D}\rangle = \rho\sum_m h_m\phi_m \quad (5)$$

Snail model. Total snail population density ($N = x + y + z$) is partitioned into three compartments: susceptible (*x*), exposed (*y*), and infected (*z*). The susceptible snail population grows at a rate (β). In most papers,[3-6,8] we used a logistic model of population growth with maximal reproduction rate, (β_0), and carrying capacity, K, in a stationary (seasonal mean) environment.

A notable exception is Gurarie, King, et al.,[15] which developed a more realistic model of resource-dependent

snail population dynamics in a highly variable (seasonal) environment.

As only susceptible and prepatent snails contribute to reproduction, we used a logistic growth-rate function

$$\beta = \beta_0 \left(1 - N / K\right)\left(x + y\right)$$

to estimate local snail numbers.

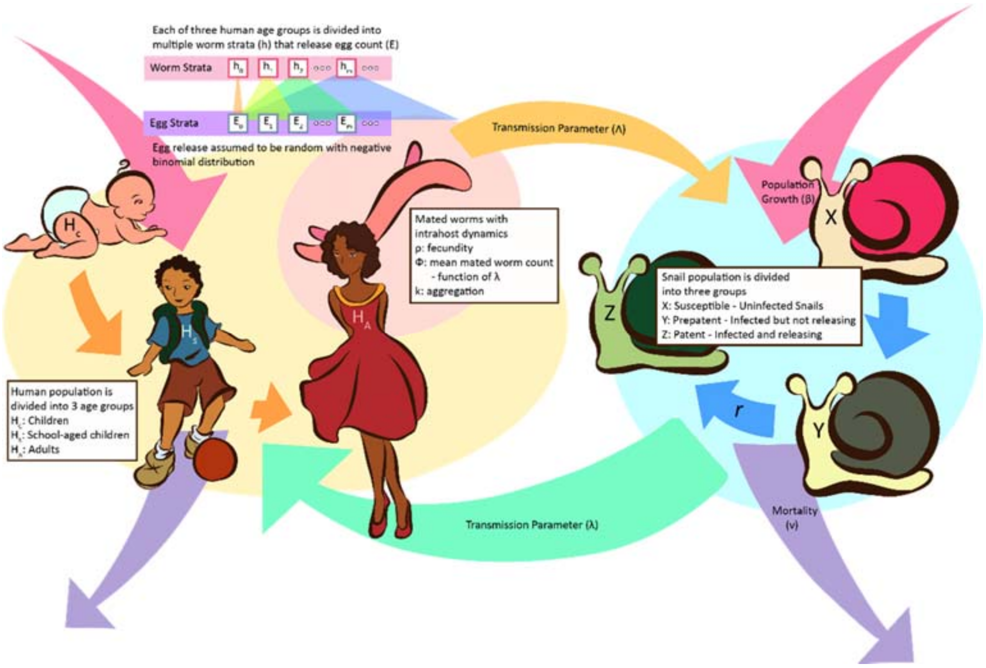

Figure A1. ***Schematic of variables in the SWB model***
Humans from three age groups (children: 0-5 year old, school-aged children: 6-14 year old, and adults: 15+) each contain a distribution of mated worms, and random egg release for each host and each burden stratum obeys a negative binomial distribution with suitable mean (= mated worm count times fecundity), and aggregation parameter k. The snail population is divided into three compartments (susceptible, prepatent, and patent) based on infection status. Transmission parameters and forces of infection (λ –snail to human, Λ- human to snail) depend on infectivity and population density of each host group.

Snail force of infection (Λ) depends on combined human infectivity (*E*) – mean egg release by the host population. Prepatent snails become cercaria-shedding snails at the patency conversion rate (r). Snails from the (*x,y*) compartments die with a natural mortality rate of *v*, while patent snails have higher mortality $\nu_1 > \nu$.

The resulting coupled system of differential equations is shown below.

$$\begin{aligned} \frac{dx}{dt} &= \beta - \Lambda x - \nu x \\ \frac{dy}{dt} &= \Lambda x - \left(r + \nu\right) x \\ \frac{dz}{dt} &= r y - \nu_1 z \end{aligned} \tag{6}$$

Human and snail infectivity and FOI in coupled system

Human and snail FOI (λ, Λ) depend on infectivity of each host, its population density (H – human, N – snail), and age-specific contact (exposure/contamination) rates ω. Combined human infectivity is a weighted sum of functions (5), with demographic population fractions $H_C + H_S + H_A = 1$, and relative contact rates ω_C (child/SAC) and ω_A (adult/SAC).

$$E = H_C \omega_C E_C + H_S E_S + H_A \omega_A E_A \tag{7}$$

We used SAC as a reference group, and included its contact rate into transmission coefficient *b* (below). Snail infectivity is represented by patent snail density $z(t)$.

The two FOI have different functional forms-- while human $\lambda_C; \lambda_S; \lambda_A$ are proportional (linear functions) of *z*, snail FOI was found by Gurarie, Lo, et al.[4] to best fit a non-linear (saturated) function,

$$\Lambda(E) = \Lambda_0 N \left(1 - \exp\left[\frac{b\, H\, E}{N} \right]^{-} \right) \tag{8}$$

The coefficient Λ_0 is the maximal invasion rate of snails by miracidia, H, N - human/snail population densities per unit habitat.

The human and snail equations are then coupled via two transmission parameters with coefficients A (snail-to-human) and B (human-to-snail). Estimated model parameters include age-specific human FOI (λ_i), worm fecundity (ρ_i), aggregation (k_i), and the resulting transmission rates (A_i, B).

Drug treatment

The drug praziquantel kills a significant fraction of adult worms (75%-85%) over short time duration. In our MDA model simulations we view it as instantaneous event, whereby SWB system is reinitialized (via reshuffling the worm burden variables, $\{h_m\}$, from higher to lower strata), in a manner determined by drug efficacy and coverage f.[1,3-5,10,12,16] The basic MDA inputs are (i) population coverage fraction ($0 < f < 1$), (ii) drug efficacy (ε - fraction of surviving worms), (iii) frequency of implementation for repeated regimens (annual, biannual, etc.).

Model Calibration and Sensitivity – Effects of Uncertainty

As our model incorporates both human and snail components of the coupled system, its calibration proceeds in two steps. 1) From the egg output or circulating antigen test data, we are able to generate a posterior distribution of likely parameter choices on the human side. (2) To calibrate transmission coefficients (snail-to-human and human-to snail) we then combine the human calibration of step (1) with the available

environmental variables, such as snail population counts, its prepatent/patent fractions, along with human snail contact rates (exposure/contamination).

In the absence of specific environmental data, we sample a broad range of possible environmental inputs values and generate ensembles of virtual communities and environments. Thus, there are two types of model uncertainties that we exploit in our analysis, i) posterior uncertainty of the calibration step, and ii) environmental uncertainty. The latter can be narrowed if additional snail information is available. In the absence of such information we explore a range of possible snail inputs consistent with 'known' human infection values.

A model's uncertainties (human + snail environment) can affect its predicted outcomes (see Figure 1A). Indeed, a prescribed human endemic infection level can produce a wide range of possible outcomes for a given treatment strategy. In applications to control analysis,[3,5-8,10,17] we employed such uncertainties to estimate the probability of obtaining a specific targeted reduction for reaching public health goals.

Figure 2A shows analysis of multi-year mass drug administration (MDA), based on a proposed modification of WHO guidelines, as we have proposed in Li, et al.[6] However, after a program is stopped (upon reaching control targets), we see that the infection is likely to rebound to near pre-control levels over a 5-year period.

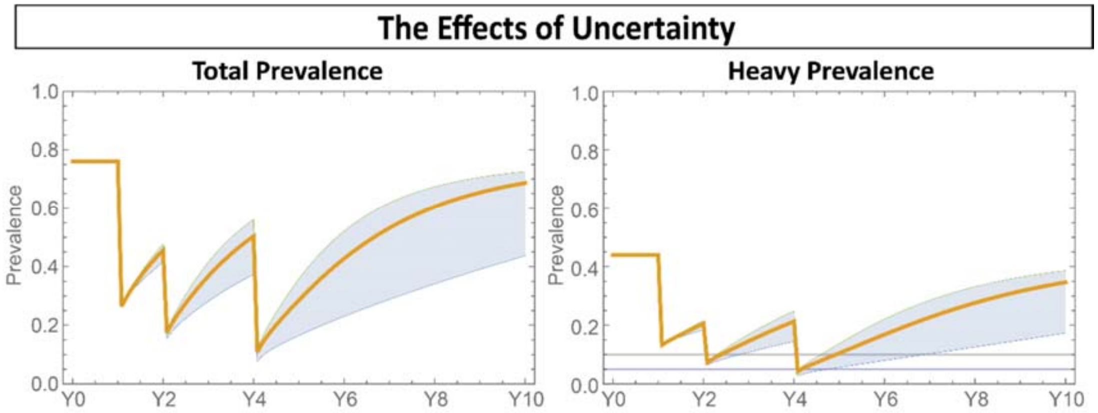

*Figure 1A . Illustration of combined (***environmental+ posterior) uncertainty for a hypothetical MDA programs with interventions on Y1-Y2-Y4. A wide range of outcomes ensues despite a single endemic initial state of human infection.**

Future Maintenance Strategy

Although the modified guidelines mentioned above are predicted to be more efficient and effective at reaching current WHO targets for 'morbidity control' and 'elimination as a public health problem', further work needs to be done regarding disease control *after* these goals have been reached. If all treatment ceases, our model predicts that rebound to initial prevalence values can happen within five years (Figure 2A). As shown in Li et al.,[6] mixed control methods such as the addition of snail control may slow the rebound. However, further operational research into the most effective strategy will need to be performed.

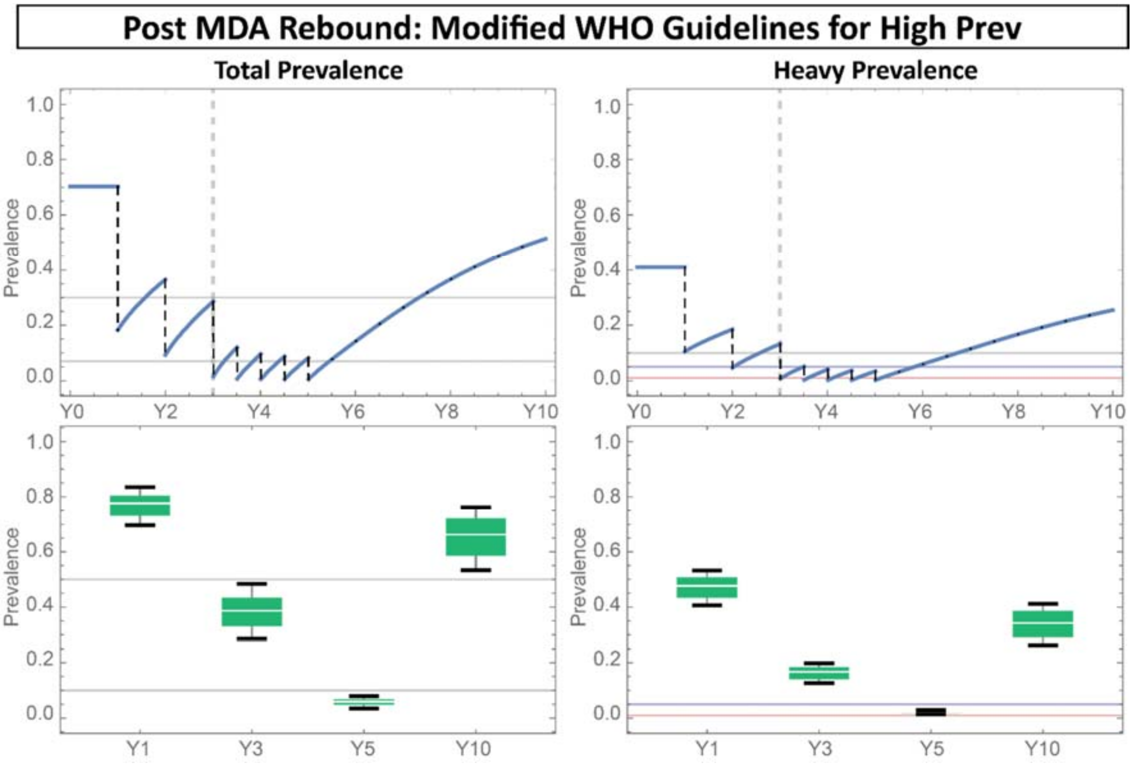

Figure 2A**. *Illustration of prediction uncertainty and prevalence rebound after MDA is stopped.***
Although elimination as a public health problem and morbidity control can be achieved for many villages in 5 years, rebound to near initial prevalence levels is predicted to occur by year 10 if no further MDA cycles are performed. 2020 goals of morbidity reduction (< 5% prevalence of heavy infection among SAC) and elimination as a public health problem (< 1% prevalence of heavy infection among SAC) goals are displayed by the blue and red horizontal lines, respectively, in the right-hand panels for heavy prevalence.

References

1. Gurarie D, King CH, Wang X. A new approach to modelling schistosomiasis transmission based on stratified worm burden. *Parasitology.* 2010;137(13):1951-1965.
2. Gurarie D, King CH. Population biology of Schistosoma mating, aggregation, and transmission breakpoints: more reliable model analysis for the end-game in communities at risk. *PLoS One.* 2014;9(12):e115875.
3. Gurarie D, King CH, Yoon N, Li E. Refined stratified-worm-burden models that incorporate specific biological features of human and snail hosts provide better estimates of Schistosoma diagnosis, transmission, and control. *Parasit Vectors.* 2016;9(1):428.
4. Gurarie D, Lo NC, Ndeffo-Mbah ML, Durham DP, King CH. The human-snail transmission environment shapes long term schistosomiasis control outcomes: Implications for improving the accuracy of predictive modeling. *PLoS Negl Trop Dis.* 2018;12(5):e0006514.
5. Lo NC, Gurarie D, Yoon N, et al. Impact and cost-effectiveness of snail control to achieve disease control targets for schistosomiasis. *Proc Natl Acad Sci U S A.* 2018;115(4):E584-E591.
6. Li EY, Gurarie D, Lo NC, Zhu X, King CH. Improving public health control of schistosomiasis with a modified WHO strategy: a model-based comparison study. *The Lancet Global Health.* 2019;7(10):e1414-e1422.
7. Toor J, Alsallaq R, Truscott J, et al. Are we on our way to achieving the 2020 goals for schistosomiasis morbidity control using current WHO guidelines? *Clinical Infectious Diseases.* 2018;to appear.
8. Truscott J, Gurarie D, Alsallaq R, et al. A comparison of two mathematical models of the impact of mass drug administration on the transmission and control of schistosomiasis. *Epidemics.* 2017;18:29-37.
9. Alsallaq RA, Gurarie D, Ndeffo Mbah M, Galvani A, King C. Quantitative assessment of the impact of partially protective anti-schistosomiasis vaccines. *PLoS Negl Trop Dis.* 2017;11(4):e0005544.
10. Gurarie D, Yoon N, Li E, et al. Modelling control of *Schistosoma haematobium* infection: predictions of the long-term impact of mass drug administration in Africa. *Parasit Vectors.* 2015;8(1):529.
11. May RM. Togetherness among schistosomes: its effects on the dynamics of the infection. *Mathematical biosciences.* 1977;35(3):301-343.
12. Wang X, Gurarie D, Mungai PL, Muchiri EM, Kitron U, King CH. Projecting the long-term impact of school- or community-based mass-treatment interventions for control of Schistosoma infection. *PLoS Negl Trop Dis.* 2012;6(11):e1903.
13. de Vlas SJ, Gryseels B. Underestimation of *Schistosoma mansoni* prevalences. *Parasitology today (Personal ed.* 1992;8(8):274-277.
14. Hubbard A, Liang S, Maszle D, Qiu D, Gu X, Spear R. Estimating the distribution of worm burden and egg excretion of Schistosoma japonicum by risk group in Sichuan Province, China. *Parasitology.* 2002;125(3):221-231.
15. Gurarie D, King C, Yoon N, Wang X, Alsallaq R. Seasonal dynamics of snail populations in coastal Kenya: Model calibration and snail control. *Advances in Water Resources.* 2017;108:397-405.
16. Li E, Gurarie D, Lo NC, Zhu X, King CH. Improving public health control of schistosomiasis with a modified WHO strategy: a model-based comparison study. *The Lancet Global Health.* 2019;to appear.
17. Hollingsworth TD, Adams ER, Anderson RM, et al. Quantitative analyses and modelling to support achievement of the 2020 goals for nine neglected tropical diseases. *Parasites & vectors.* 2015;8(1):1-28.
